# Memory of Natural Killer Cells: A New Chance against *Mycobacterium tuberculosis*?

**DOI:** 10.3389/fimmu.2017.00967

**Published:** 2017-08-14

**Authors:** José Alberto Choreño Parra, Nayeli Martínez Zúñiga, Luis Antonio Jiménez Zamudio, Luis Armando Jiménez Álvarez, Citlaltepetl Salinas Lara, Joaquín Zúñiga

**Affiliations:** ^1^Laboratory of Clinical Immunology I, Escuela Nacional de Ciencias Biológicas, Instituto Politécnico Nacional, Mexico City, Mexico; ^2^Laboratory of Immunobiology and Genetics, Instituto Nacional de Enfermedades Respiratorias “Ismael Cosío Villegas, Mexico City, Mexico; ^3^Brain Bank, Department of National Laboratories of Experimental Services, Centro de Investigación y de Estudios Avanzados (CINVESTAV), Instituto Politécnico Nacional, Mexico City, Mexico; ^4^Department of Pathology, Instituto Nacional de Neurología y Neurocirugía “Manuel Velasco Suárez”, Mexico City, Mexico

**Keywords:** natural killer cells, innate memory, memory-like natural killer cells, *Mycobacterium tuberculosis*, innate immunity

## Abstract

Natural killer (NK) cells are lymphocytes of the innate immune system, which play an important role in the initial defense against a wide variety of pathogens, including viruses and intracellular bacteria. NK cells produce cytokines that enhance immune responses directed toward pathogens and also exert cytotoxic activity against infected cells, thereby eliminating the reservoir of infection. Their role in defense against *Mycobacterium tuberculosis* (*Mtb*) has been recently studied, and there is increasing evidence that highlight the importance of NK cell function during pulmonary tuberculosis (PTB), especially in the absence of optimal T-cell responses. Additionally, in the last years, it has been observed that NK cells mediate secondary responses against antigens to which they were previously exposed, an ability classically attributed to lymphocytes of the adaptive branch of immunity. This phenomenon, called “innate memory,” could have important implications in the efforts to develop therapies and vaccines to improve the initial phases of immune reactions against different microorganisms, especially those to which there is not yet available vaccines to prevent infection, as is the case for tuberculosis. Therefore, the possibility of inducing memory-like NK cells ready to act prior to contact with *Mtb* or during the earliest stages of infection becomes quite interesting. However, our understanding of the mechanisms of innate memory remains incomplete. Here, we review recent literature about the mechanisms involved in the formation and maintenance of NK cell memory and the role of these cells in the immune response during tuberculosis. Finally, we discuss if the current evidence is sufficient to substantiate that NK cells exert more rapid and robust secondary responses after consecutive encounters with *Mtb*.

## Introduction

Natural killer (NK) cells are innate lymphocytes with cytotoxic activity that cannot be classified within T- and B-cell lineages, as they differ from these lymphocytes in expression of CD56 (NCAM) and a lack of CD3 and CD19 (1). NK cells are part of group 1 of innate lymphoid cells (ILC1) (2) and were originally referred to as “natural killers” due to their intrinsic ability to induce lysis of target cells without previous antigen exposure (3, 4). Although NK cells use non-antigen specific mechanisms to exert effector functions, a growing body of evidence supports the idea that NK cells can exert recall responses against previously recognized antigens (5). This phenomenon, referred to as “innate memory,” remained unnoticed for a long time as NK cells are constitutively “ready to act” and were not thought to improve their effector capacity after repeated antigenic exposure. In contrast to this belief, multiple studies have demonstrated that NK cells become more efficient during secondary responses to viruses, haptens, and intracellular bacteria (6–12). Nonetheless, the evidence of secondary responses mediated by NK cells against mycobacteria remains controversial.

Mycobacteria are pathogens that have co-evolved with and caused infection in mammals over thousands of years, resulting in the development of many strategies to evade protective innate and adaptive immune responses (13, 14). As NK cells play a role in host defense against *Mycobacterium tuberculosis* (*Mtb*) and there is increasing evidence of the importance of NK cell function during pulmonary tuberculosis (PTB), the possibility that NK cells may develop more efficient responses upon secondary exposure to *Mtb* becomes an interesting question that needs to be experimentally explored. If NK cells were found to be more effective upon secondary exposure to antigen, this aspect of NK cell biology could be manipulated for clinical application. For example, exposure of NK cells to antigens from different strains of *Mtb* would be helpful in improving vaccine efficacy and perhaps enhancing innate immune responses against *Mtb* in the clinical landscape of tuberculosis pathogenesis. However, the mechanisms responsible for induction, maintenance, and regulation of memory-like NK cells specific for *Mtb* must be extensively investigated using *in vitro* assays, experimental animal models, and eventually in human clinical studies. In this review, we focus on the evidence generated during the last decade about NK cell memory and critically discuss the experimental data which support the hypothesis that NK cells exert more rapid and robust secondary responses after consecutive encounters with *Mtb*.

## Biology of NK Cells

Natural killer cells represent 5–15% of circulating lymphocytes in peripheral blood and their half-life, both in humans and mice, is about 2 weeks (15). They can be found in the spleen, liver, lung, thymus, uterus, lymph nodes, and they are also recruited to locally-inflamed peripheral tissues (16). Their “homing” under normal conditions depends on the expression of chemokine receptors which guide them to various sites (17, 18).

Natural killer cell function is determined by a balance between signals from both activating and inhibitory membrane receptors. These receptors are germline encoded and do not undergo somatic rearrangement like T-cell receptors. Hence, these cells have a limited repertoire, which allow them to recognize a reduced number of ligands, many of which are related to molecules of the major histocompatibility complex class I (MHC-I) (19, 20). Induction and maintenance of NK cell function is determined by NK cell receptors binding to their respective ligand, leading to subsequent activation of intracellular tyrosine-based activating motifs or intracellular tyrosine-based inhibitory motifs (ITIM), resulting in an activating or inhibitory effect, respectively (21, 22). Under homeostatic conditions, upon NK cell receptor binding of MHC-I expressed by normal (i.e., non-tumorigenic or uninfected) cells, SHP-1 and SHP-2 protein tyrosine phosphatases are recruited to ITIM present in the intracellular domain of inhibitory NK cell receptors. This results in the arrest of tyrosine kinase-based activation signals, and no further action is taken (20). These inhibitory NK cell receptors belong to four different families of membrane-bound receptors, which recognize MHC-I molecules: the killer-cell immunoglobulin-like receptors (KIR), the immunoglobulin-like transcripts (also known as leukocyte immunoglobulin-like receptors, or CD85), the C-type lectin-like receptors (CLR) of the Natural Killer Group 2 [NKG2 heterodimers (i.e., CD94/NKG2A)], and the killer-cell lectin-like receptor subfamily A (also known as Ly49) (21, 23).

Alternatively, when mature NK cells interact with target cells expressing an abnormal or diminished level of MHC-I (such as in the case of viral infection), SHP-1 and SHP-2 are not recruited and activation signals mediated through the NK cell activating receptors are not suppressed. This leads to a predominance of activating stimuli, triggering the cytotoxic effector function of NK cells and subsequent target cell death, which promotes clearance of the infected cells. NK cells express a large range of activating receptors, which can be classified into four groups based on function and ligand interaction. First, they express the low affinity activating Fc receptor, CD16a (also known as FcγRIIIa), which recognizes the Fc region of immunoglobulin 1 (IgG1) and 3 (IgG3) and mediates antibody-dependent cellular cytotoxicity (ADCC). Second is the expression of natural cytotoxicity receptors (i.e., NKp30, NKp44, and NKp46), which belong to the immunoglobulin superfamily. The last groups of activating receptors are of the same families as some of the inhibitory receptors: the CLR-NKG2 family (i.e., NKG2D) and the KIR family. These two families of receptors induce cytokine production and cytotoxic activity after binding by MHC-I-associated molecules expressed on the surface of infected or damaged cells (i.e., ULBP, MIC-A, MIC-B, RAE1, H60, and MULT1) (23) and viral proteins that are MHC-I-like [i.e., m157 encoded by mouse cytomegalovirus (MCMV)] (24). Moreover, NK cells can directly recognize microorganisms through the interaction of Toll-like receptors (TLR) with pathogen-associated molecular patterns expressed by the pathogen (25, 26).

In addition to the wide variability of receptors that regulate NK cell activity, there are several subpopulations of NK cells with different degrees of maturation, and hence, with diverse functional profiles. In mice, the extent of expression of CD11b and CD27 discriminates between subsets of different maturity. CD11b^low^ CD27^+^ cells are present in bone marrow and lymph nodes and have a more immature phenotype, while CD11b^+^ CD27^low^ NK cells localized in blood, spleen, liver, and lung have cytotoxic capacity, the ability to produce cytokines in response to stimulation, and decreased replicative potential (27). In humans, NK cells are distinguished by the expression of CD16 and varying levels of expression of CD56, which can be used to discriminate different subpopulations of NK cells. Those cells expressing low levels of CD56, or CD56^dim^CD16^+^, are mature, differentiated cells in the periphery with high cytotoxic activity. The CD56^bright^CD16^−^ NK cells are associated with a lower level of development and poor lytic function and produce cytokines when stimulated with IL-12, IL-15, and IL-18 (28). These subpopulations are frequently located within lymph nodes, sites which have been considered centers of production and maturation of NK cells (such as the thymus is for T cells), and required for the development of NK cell “self-tolerance” (29–33).

Although much of the role of NK cells in tumor surveillance and in the defense to infectious agents is related with their cytotoxic properties, cytokine production is also a prominent function that places these cells as key orchestrators and regulators of innate and adaptive immunities. Along with IFN-γ and TNF-α, the main NK cells-derived cytokines that exert proinflammatory effects on other hematopoietic and non-hematopoietic cells in response to infection (34, 35), there is a growing list of soluble mediators that can also be released after activation of NK cells in different settings, including regulatory IL-5, IL-10, IL-13 (36–38), pro-inflammatory IL-17 (39), epithelial regenerative and protective IL-22 (40, 41), the growth factor GM-CSF (42), as well as chemokines MIP-1α, MIP-1β, IL-8, and RANTES (43–46). Such ability is triggered principally in response to other exogenous cytokines that supports the proliferation and function of NK cells (47), but as occurs with the cytotoxic activity, recognition of membrane-bound ligands on target cells also regulates the production of different mediators by certain NK cell subsets in humans (48). Moreover, NK cells can produce IL-2 to promote their proper activation after clustering in multicellular groups and come into cell-to-cell homotypic interactions (49).

## Role of NK Cells in the Immune Response Against *M. tuberculosis*

Natural killer cells play an important role in the immune response against viruses and intracellular bacteria due to their ability to kill infected cells and to eliminate intracellular pathogen reservoirs (50). There are several examples in which NK cell activity is crucial for disease control; possibly, one of the most well-documented examples is the role of NK cells during cytomegalovirus (CMV) infection (34, 50–54). However, the participation of NK cells in the immune response during PTB has been underappreciated, despite the fact that NK cells are an important source of IFN-γ, a cytokine vital to the immune response against *Mtb* through activation of macrophages, and subsequent enhancement of bactericidal activity (55). Furthermore, NK cells produce IL-22, a cytokine that has been shown to have a protective role during chronic stages of infection by emergent hyper-virulent strains of *Mtb* (56, 57).

In spite of the historic lack of attention received by NK cells during *Mtb*-associated immunity, mounting evidence suggests that NK cell function is important during PTB. First, several studies in humans have revealed that the risk of developing the active form of disease is related with the phenotype and functional state of NK cells isolated from peripheral blood. In fact, there is a higher prevalence of the KIR group A haplotype (expressing more inhibitory KIR) among patients with active tuberculosis when compared with resistant individuals. Indeed, certain KIR genes have increased correlation with the likelihood to become ill (i.e., KIR3DL1, KIR2DL3, KIR2DS1, and KIR2DS5), whereas others associate with a protective phenotype (i.e., KIR3DS1, KIR2DS2) (58–61). Portevin et al. confirmed these observations by demonstrating NK cells from healthy individuals’ respond to direct contact with *Mtb* and *M. bovis* BCG and that the degree of such responses was dependent on the KIR haplotype. Also, they observed that NK cells are recruited into the lung lesions of patients with chronic *Mtb* infection (62).

When cultured in the presence of live *Mtb* bacilli, both subpopulations of human NK cells (CD56^bright^ and CD56^dim^) respond and exert effector functions (63). However, in patients with active PTB, there is reduced frequency of CD56^bright^ cells in the peripheral leukocyte population, accompanied by decreased expression of NK cell activating receptors (NKp30, NKp46), resulting in declined functional capacity (64, 65). This functional impairment of NK cells is also associated with an increase in CD4^+^ CD25^+^ regulatory T cells (T_reg_), which may regulate the activity of NK cells (66). It is unknown if such alterations in NK cell functionality are related to the risk of bacterial dissemination to extrapulmonary sites.

Although the evidence mentioned earlier supports a role for NK cells in the defense against *Mtb* in patients with chronic infection, it is important to remember that these cells are innate in nature and act early during microbial defense (50). Functional assessment of human NK cells during initial stages of PTB is difficult, as most patients with pulmonary disease are diagnosed long after initial contact with the bacillus. Therefore, studies in different animal models have been conducted to evaluate NK cell activity in the early phases of the immune response against tuberculosis, with contradictory results. Specifically, Feng et al. showed that during *Mtb* infection of *Rag1*^−^ deficient mice (which lack T and B cells), NK cells drive the immune response and resistance against PTB by secreting IFN-γ (67). In contrast, when *Mtb* infection occurs in T- and B-cell sufficient animals, NK cell depletion does not influence *Mtb* bacterial burden in the lungs or disease severity (68). This is in agreement with the evidence, both in mice and humans, of the redundancy of ILC activity in the context of a complete adaptive immune system (69, 70). Despite the perhaps redundant role NK cells play during tuberculosis in immunocompetent individuals, their role in protection against *Mtb* could be of particular relevance in cases of T-cell dysfunction, i.e., in individuals infected with human immunodeficiency virus (HIV). This concept is of great importance, as HIV/AIDS is the leading comorbidity in *Mtb*-infected individuals, and according to the 2015 WHO Tuberculosis report, 35% of HIV-related deaths were due to PTB (71).

Recent data suggest that NK cells may modulate the development of mycobacteria-specific adaptive immune responses. In this regard, Vankayalapati et al. demonstrated that NK cells isolated from human peripheral blood can regulate effector functions of CD8^+^ T cells in response to *Mtb* infection. NK cell depletion reduced the frequency of CD8^+^ IFN-γ^+^ T cells and decreased their capacity to lyse infected macrophages after *in vitro* exposure to *Mtb* bacilli (72). In addition, NK cells induced lysis of expanded CD4^+^ CD25^+^ T_reg_ after incubation with *Mtb*-infected monocytes; this lysis was shown to be dependent on NK cell NKG2D recognition of ULBP-1 on the T_reg_ surface (73).

*In vitro* assays have also revealed specific interactions between NK cells with infected phagocytes as well as with bacilli in their extracellular form. Specifically, it was shown that NK cells efficiently recognize certain *Mtb* and *M. bovis* BCG cell wall components through TLR-2 and NKp44 receptors (26, 74, 75). Of note, NKG2D and NKp46 activating receptors are ligated by ULBP-1 and vimentin, respectively, whose expression increases on the surface of macrophages that have ingested bacteria (76–78). After recognition of these ligands, NK cells release IFN-γ and IL-22, increasing the bactericidal capacity of infected phagocytes and may perform cytotoxic measures against these infected cells to eliminate intracellular pathogen niches (79, 80). Moreover, activated NK cells kill extracellular mycobacterial bacilli by releasing perforin and granulysin, *via* a mechanism dependent on intracellular signaling pathways mediated by kinases such as ERK, JNK, and p38MAPK (81). Production of proinflammatory cytokines, specifically IFN-γ, also may be triggered by direct contact with *Mtb* antigen; this activity is enhanced by cell-to-cell interaction with dendritic cells and associated IL-12 signaling (82).

Finally, NK cells play a role during the development of protective immunity conferred by vaccination. Murine exposure to BCG was shown to increase the number of IL-22-producing NK cells, and NK cell depletion induced an expansion of CD4^+^ CD25^+^ T_reg_ during re-challenge with *Mtb* H37Rv as well as increased bacterial burden and diminished T-cell responses (83).

## Immunological Memory Mediated by NK Cells

Natural killer cells are classically categorized as members of the innate immune system, as it was thought that repeated encounter with antigen did not augment or enhance their effector functions. Notably, in 2006, Leary et al. demonstrated that NK cells had adaptive memory qualities by using a mouse model of hapten-induced contact hypersensitivity (CHS) (5). In the absence of both T and B cells, NK cells were sufficient to mediate CHS responses in a hapten-specific manner, as CHS only occurred in those instances where the hapten used during challenge was the same hapten used during sensitization. This study also supplied a more structured understanding of NK cell function and delineated the mechanisms NK cells use to circulate within lymphatic vessels and access lymph nodes, where antigen priming occurs. Once NK cells have been activated in secondary lymphoid organs, they migrate to the liver and reside there until antigen re-exposure, then home to sites of antigenic challenge. Moreover, transfer of liver-resident and not spleen-resident memory-like NK cells into healthy mice resulted in transfer of the hypersensitivity phenotype (5). Further analyses revealed later that liver NK cells with the ability to exert secondary responses to haptens belong to a specific subset (denoted by CD49a^+^ DX5^−^) and that recall responses to subsequent contacts with haptens are dependent on the activity of NLRP3 inflammasome (84, 85).

Sun et al. subsequently found that MCMV infection elicited an immune response mediated by NK cells, which emulated all the hallmarks of a traditional memory response (7), characterized by an initial proliferation phase with clonal expansion of NK cells expressing the Ly49H receptor (which recognizes the m157 protein of the MCMV). This clonal expansion was dependent on Ly49H/DAMP12 signaling (receptor ligation, or “Signal 1”) and IL-12 priming through STAT-4 activity (cytokine help or “Signal 2”), as well as co-stimulation by DNAX accessory molecule 1 (co-stimulation or “Signal 3”) (7, 86). After this proliferation phase, contraction of effector NK cells occurred by mitochondrial apoptosis and was followed by the development of a pool of memory cells, which survived cell death through mechanisms of mitophagy (87). Similar to T-cells (88), NK cells required IL-15 signaling as well as the regulatory activity of miR155 on Noxa and SOCS1 for optimal differentiation into memory-like NK cells (89, 90). This pool of memory-like NK cells resided in the spleen and other lymphoid and non-lymphoid organs. Interestingly, transfer of MCMV-memory-like NK cells into neonate mice resulted in protection from a lethal challenge of MCMV (7).

Similar to the findings in MCMV-infected mice, clonal expansion of CD94/NKG2C^+^ NK cells was observed in humans seropositive for human cytomegalovirus (91–93) as well as in transplant recipients who either underwent CMV reactivation or were seronegative and had received organs from seropositive donors (94, 95). These data show that transfer of NK cells with long-lasting survival and enhanced antigen-specific activity is a viable immune mechanism in humans as well as mice. Clonal NK cell expansion in humans was also observed in response to other viral infections, such as hantavirus, chikungunya, hepatitis B virus, hepatitis C virus, and HIV (96–100). Further evidence of memory and clonal expansion of NK cells in response to viral infection was found using mouse models of immunization with antigens of genital HSV-2 virus (12), influenza virus, vesicular stomatitis virus, and even HIV, a pathogen that does not infect mice naturally. Moreover, this virus-specific immune response by NK cells and the associated protection are mediated only by those NK cells isolated from the liver expressing CXCR6 (6). Interestingly, influenza vaccination of healthy humans enhanced the amount of IFN-γ produced by NK cells up to 6 months post-immunization (101). These data support that human NK cells, apart from expanding during antigen encounter, also display a better response in subsequent exposures to viral antigens, similar to that which occurs in mice. Although most of these recall responses occurred in the context of viral infection, it was recently documented that memory-like NK cells were expanded in response to infection with the intracellular bacterium *Ehrlichia muris* (9). This pathogen belongs to a genus of which it was recently reported that NK cells play an important role in the associated inflammatory responses (102). In addition, human and mouse NK cells display enhanced activity when they are primed with cytokines and later re-exposed to the same stimuli in conjunction with engagement of activating receptors by antibodies or cognate ligands (103–105).

Collectively, these studies support that immunological memory is an intrinsic property of NK cells conserved in vertebrates. Of note, NK cells have secondary responses against both previously unencountered antigens and antigens that have not represented an evolutionary pressure, as in the case of haptens and HIV infection, respectively, in murine models (5, 6). In addition, it has been observed that HIV elicits memory of long duration in macaques, a mammalian species that, as the mouse, is not the natural host of this virus (8). Nevertheless, there are several aspects of innate memory of NK cell subsets that remain poorly understood. One question is whether NK cells with the ability to mediate memory responses belong to a specific subgroup of conventional NK cells or if they are in fact lymphocytes of a different subset within the group 1 ILC? Second, what markers and patterns of expression can be used to identify these memory-like NK cells? To date, it is well established in mice that memory-like NK cells are located preferentially in the liver, but their specific phenotype has only been described in mouse models using haptens and MCMV (5, 7, 84). It will be important to address if there is a specific NK cell subset induced by certain paired NK cell receptor-ligand interactions, or associated with different classes/types of pathogens and subsequent responses.

## Evidence of Memory Against *M. tuberculosis* within NK Cell Subsets

Different groups have recently sought to identify memory responses mediated by NK cells against mycobacteria, and the results have been contradictory, proving it difficult to define the importance of human NK cells in the immune pathogenesis of tuberculosis. NK cells isolated from pleural fluid of subjects with pleural effusion associated with active PTB express the memory marker CD45RO and produce higher amounts of IFN-γ and IL-22 in response to stimulation with interleukins and *M. bovis* BCG when compared with CD45RO- cells (106, 107). As these NK cells were isolated from a specific inflamed anatomical site, it would be of great interest to assess whether peripheral and tissue resident NK cells also express CD45RO and if this expression confers enhanced effector functions against different species of mycobacteria.

Additional evidence in favor of memory against *Mtb* within NK cell subsets has been provided by Suliman et al., who found that BCG re-vaccination of subjects latently infected with *Mtb* induced long-term responses in NK cells, which persisted for up to 1 year after re-challenge (108). In this study, they also found that BCG vaccination of humans at birth induced augmented activity of NK cells in 5-week-old neonates. Another study also showed that BCG vaccination in healthy subjects promoted the production of proinflammatory cytokines, particularly IL-1β, by NK cells in response to *Mtb* and other fungal pathogens 3 months after the initial exposure (109).

Meanwhile, Kawahara et al. were unable to demonstrate enhanced secondary responses by NK cells isolated from mice vaccinated with BCG (110). Using a mouse model of immunization with BCG, this group did not find functional differences between NK cells isolated from the spleens of vaccinated and unvaccinated animals, particularly in their capacity to produce cytokines after *in vitro* exposure to *Mtb* H37Rv. Also, NK cells stimulated *in vitro* with BCG antigens did not have increased cytotoxicity against *Mtb*-infected macrophages (110). As Leary et al. had shown that the liver-resident NK cells were the subset responsible for enhanced responses (5, 111), it would have been beneficial to assess memory responses in NK cells isolated from tissues other than spleen, i.e., the liver, in these BCG vaccinated mice.

However, a recent study using an experimental model of *Mtb* infection in mice showed that BCG vaccination promoted an IL-21-dependent expansion of a CD3^−^NKp46^+^ CD27^+^ KLRG1^+^ memory-like NK cell subset residing in lymph nodes and spleen. These cells produced higher amounts of IFN-γ compared to other subsets of NK cells and conferred protective responses against *Mtb* upon transfer to unvaccinated animals. This finding was also confirmed *in vitro* using latently infected human cells: CD3^−^CD56^+^ CD27^+^ NK cells were able to reduce *Mtb* CFU when co-cultured with autologous infected macrophages more effectively than CD3^−^CD56^+^ CD27^−^ NK cells. In addition, the responses of these CD3^−^NKp46^+^ CD27^+^ KLRG1^+^ cells were specific as *in vitro* exposure to *Candida albicans* did not induce NK cell expansion, as seen with *Mtb*. This study is possibly the first evidence of the existence of a subpopulation of NK cells with the ability to mount recall responses against repeated exposures to *Mtb*, highlighting the potential usefulness of memory-like NK cells to improve the efficacy of vaccination against *Mtb* (112).

## Challenges

It is important to mention that studies in humans that observed memory-like responses in NK cells isolated from pleural fluid in the context of tuberculous pneumonia are only descriptive and did not evaluate the parameters which characterize an effective adaptive immune response. This is the same situation for the work done by Kawahara et al. (110) whose data, although debatable, cannot be ignored. The fact that certain subsets of human NK cells respond more robustly after re-exposure to several strains of mycobacteria and to activation with non-antigenic stimuli does not necessarily mean that these are memory cells, even when such cells express markers classically attributed to adaptive lymphocytes with the ability to improve their function after subsequent contacts with pathogen-derived antigens. Although they might belong to the memory-like NK cell subset supposedly induced by cytokines, which mediate secondary responses to specific and non-specific re-challenges, the exact mechanisms that trigger their development and maintenance have not been vigorously evaluated. Additionally, we cannot rule out the possibility that these CD45RO^+^ NK cells were expanded in response to other pathogens and became involved in the immune response to *Mtb* through a bystander mechanism.

At the same time, the lack of differences observed by Kawahara et al. in the splenic NK cell response to *in vitro Mtb* exposure between BCG vaccinated and unvaccinated animals does not mean that these cells do not “remember” previous antigen encounter and cannot exert secondary responses. Perhaps it is that these cells are simply not the subset capable of mounting a secondary response and it is only a matter of finding cells from an anatomical location, which harbor the effective subset, such as the liver.

Therefore, an animal model is needed, which allows detailed evaluation of clonal NK cell proliferation in response to PTB, as that which occurs in case of MCMV infection (7). However, this will be difficult to achieve as there are no sufficient mouse models of tuberculosis that strictly emulate the natural infection, which occurs in humans, and there is a lack of an NK cell receptor that can easily identify or define clonal NK cells with antigen specificity. In addition, we still have no markers to delineate exactly which subpopulations of NK cells are able to develop memory-like qualities in mice. In this regard, it will be important to identify mouse NK cell subsets currently known to expand in response to *Mtb* infection, as well as those populations which possess surface molecules implicated in previous studies with other pathogens. Also, pathogen species-difference specificity will need to be assessed to understand the breadth of memory NK cells induced upon antigenic stimulation. Moreover, this model must address whether memory-like NK cells migrate to a specific anatomic site and evaluate whether these presensitized NK cells confer protection when transferred to animals that have not previously come into contact with the pathogen. This last point is likely the most important aspect that future research should evaluate. Although the pleural effusion human studies support the existence of NK cell memory, they also call into question whether memory responses mediated by NK cells play a protective or pathogenic role, as the patients in this study developed severe disease complications. Moreover, although Suliman et al. observed that vaccination of neonates with BCG at birth induced enhanced responses in NK cells 5 weeks later (108), long-term follow-up is needed to find out if these responses persist indefinitely and whether they influence the risk of active PTB in the future, independently of the induction and activity of memory T cells.

In this regard, the study performed by Vankayalapati and colleagues utilizes almost all of the features that we propose an experimental model must possess to characterize immune memory mediated by NK cells in the context of *Mtb* infection. However, it would be of great interest to address if there is expansion of additional subsets of memory-like NK cells in tissues other than spleen and lymph nodes (i.e., the liver) and if such cells confer protection against *Mtb* infection. Also, other mechanisms regulating the proliferation and differentiation of memory-like NK cells must be evaluated, including a broader spectrum of cytokines that might be involved in the expansion of CD3^−^NKp46^+^ CD27^+^ KLRG1^+^ NK cells as well as other subpopulations with distinct phenotypes. Moreover, further analysis is needed to reveal the receptors and *Mtb*-derived antigens implicated in the activation and expansion of memory-like NK cells. Use of blocking antibodies and/or knockout animals lacking different NK cell membrane receptors would allow further study of these mechanisms. Finally, to determine whether expanded NK cell subsets protect from disease in humans, a comparative study is required that includes, in addition to subjects latently infected with *Mtb*, both individuals with active PTB and those with disseminated disease.

**Figure 1:**
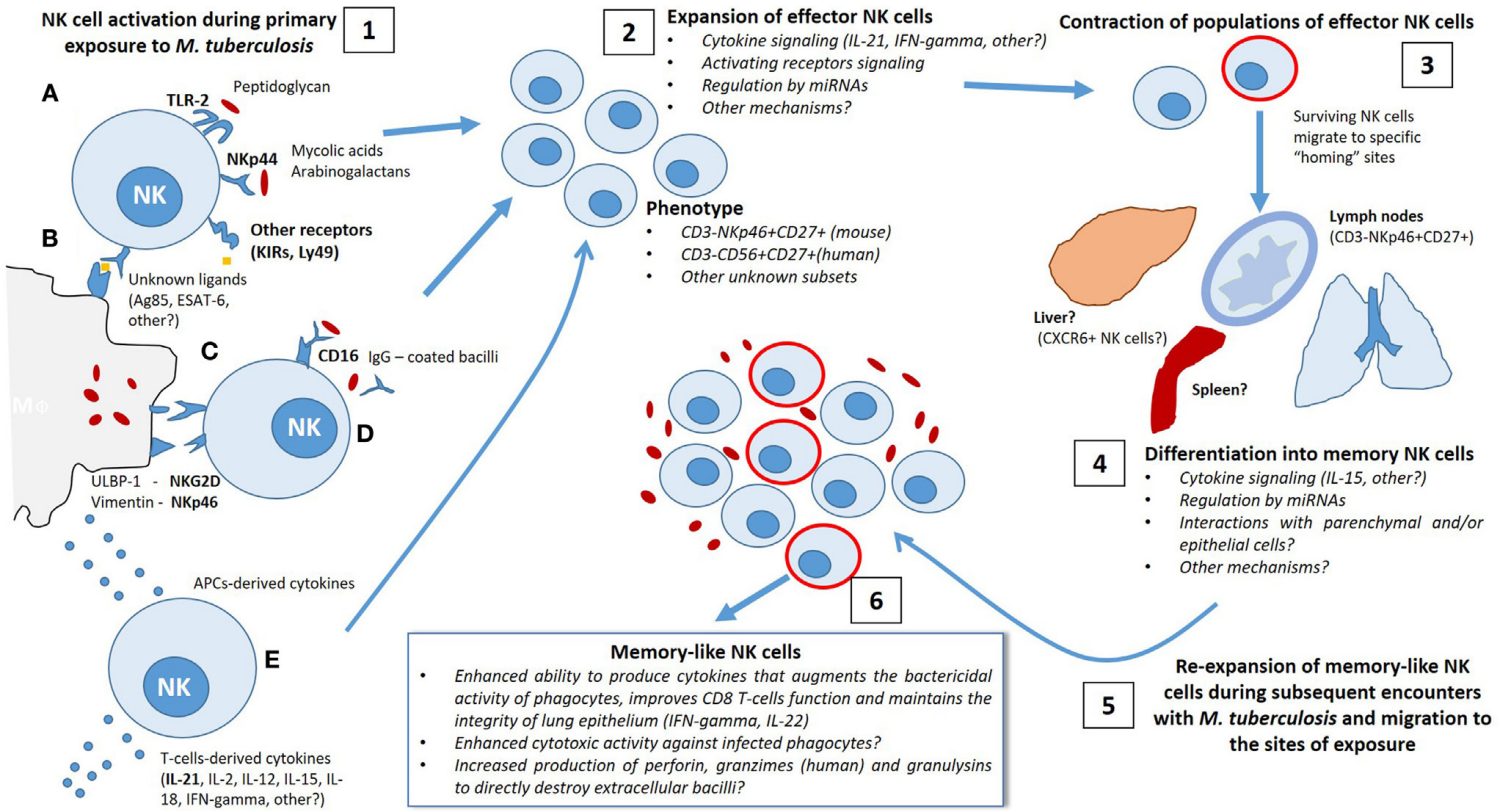
Possible mechanisms implicated in the differentiation of memory-like natural killer (NK) cells during the immune response elicited by *Mycobacterium tuberculosis* (*Mtb*) infection. 1. After primary exposure to *Mtb*, either by natural infection or vaccination, NK cells become activated by distinct antigenic and non-antigenic stimuli, allowing involvement in the initial defense against the bacillus. Direct antigenic stimulation may be provided by **(A)** direct interaction of *Mtb*-derived antigens (i.e., peptidoglycan, mycolic acids, arabinogalactan and other unknown ligands) with activating NK receptors or by Toll-like receptors engagement or **(B)** NK receptor binding to antigen presented in the context of major histocompatibility complex class I expressed on the surface of infected macrophages and/or dendritic cells. Non-specific activation of NK cells could be induced by **(C)** interaction with danger signals expressed at the membrane of infected phagocytes, such as Vimentin/NKp46 interactions, or **(D)** CD16-mediated antibody-dependent cellular cytotoxicity triggered by binding to the Fc regions of opsonized IgG-coated bacilli, as well as **(E)** priming with macrophage-derived and T-cell-derived cytokines. 2. Activation of NK cells elicits a rapid expansion of effector clones with an specific phenotype (CD3^−^NKp46^+^ CD27^+^ KLRG1^+^, others) in a cytokine-dependent manner (IL-21, IFN-γ, others), which may require additional mechanisms of regulation, potentially by miRNA. 3. Following the initial phase of proliferation, there is a contraction of effector NK cell population and the surviving cells become long-lived and migrate to specific “homing” sites to differentiate into memory-like NK cells. 4. It is possible that certain tissues contain the specific requirements needed by different subpopulations of NK cells to improve their function and to exert secondary responses. 5–6. Subsequent encounters with *Mtb* trigger a re-expansion of several subsets of memory-like NK cells with enhanced ability to respond to antigenic and non-antigenic stimuli depending on the signals received during the first mycobacterial exposure.

In Figure 1, we show the possible signals involved in induction of NK cell secondary responses by several specific and non-specific stimuli. We also highlight the mechanisms implicated in the differentiation of a subset of memory NK cells capable of responding more efficiently during repeated contact with *Mtb*.

## Arguments in Favor of the Existence of NK Cell Memory Against *M. tuberculosis*

The fact that NK cell responses against haptens, viruses, and intracellular bacteria are characterized by hallmarks of adaptive immunological memory supports the hypothesis that NK cells can become sensitized and memory like in response to repeated exposure to *Mtb*. Despite current controversial evidence supporting the existence of memory-like NK cells and their role in the immune pathogenesis of tuberculosis and that these findings must be confirmed in experimental animal models and in humans, we consider there are at least two reasonable arguments to support this assertion. First, some of the studies that defined memory-like qualities of NK cells were performed using animal models with infection by pathogens to which it is still unclear whether NK cells play an important role in the defense against them (6, 8). Second, this memory-like quality of NK cells was identified using a model of delayed hypersensitivity induced by haptens (5) and there is little evidence to support why NK cells would have need to “remember” a hapten encounter. Thus, it would appear that memory is an intrinsic characteristic of NK cells, independent of the target antigen.

Although they do not possess antigen receptors generated by genetic rearrangement, NK cells do have receptors which allow direct antigenic contact, resulting in subsequent cellular activation (74, 75). Of note, while direct contact with target antigen is an important step in the generation of memory T cells, this step is not necessary for the development of memory-like NK cells. NK cells also undergo secondary responses following activation by cytokines (103, 104). In the context of proinflammatory environments found at the sites of infection, this cytokine stimulation may augment NK cell function, as is seen when CD8^+^ T cells differentiate into antigen-non-specific memory cells after being activated with cytokines through a “bystander” mechanism (113).

Finally, species of the mycobacterium complex have infected hominids for the past 3 million years and have supplied a constant evolutionary pressure for innate and adaptive cells of the mammalian immune system (13, 14). There is evidence of immunological memory existing prior to the emergence of lymphocytes (114, 115), and NK cells, as well as their progenitors, are more ancient with respect to the cells of the adaptive immune system (116). Therefore, during mammalian evolution, there has been ample time for NK cells to learn from contact with these ancient pathogens and modify responses against them, but, at the same time, *Mtb* may have acquired evolutionary advantages to evade such responses (117).

## Conclusion and Perspectives

**Figure 2:**
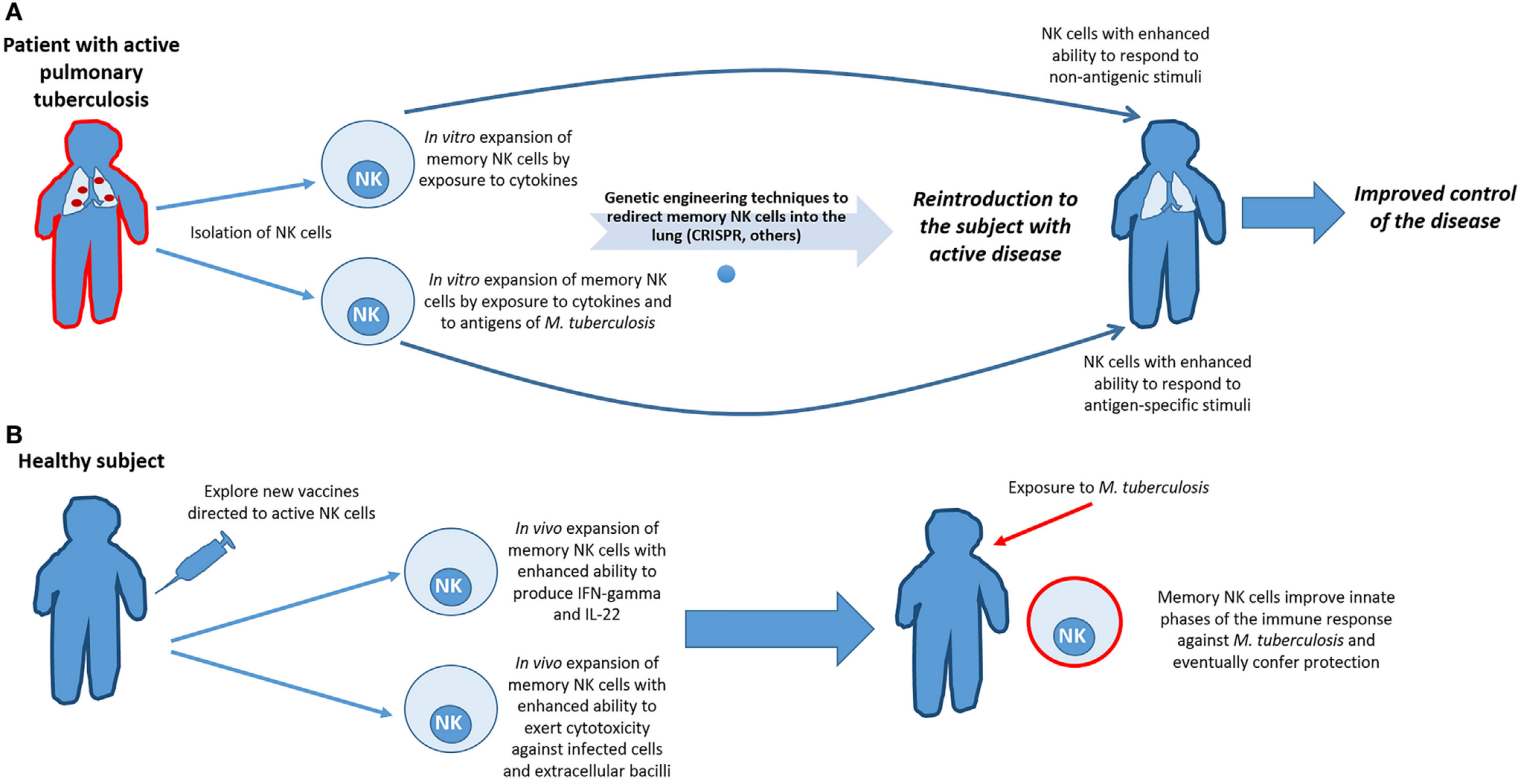
Harnessing the adaptive features of natural killer (NK) cells to improve the clinical evolution of tuberculosis and to augment vaccine efficacy. **(A)** In patients with active pulmonary tuberculosis, and even in those latently infected, NK cells could be isolated and primed through *in vitro* exposure to cytokines and/or *Mycobacterium tuberculosis* (*Mtb*) antigens, in order to expand subsets of memory-like NK cells with the enhanced ability to respond to antigenic and non-antigenic stimuli after recurrent bacterial encounters. Redirecting them to the sites of exposure would be benefit from the use of techniques of genetic engineering currently available. However, due of the cost of such proceedings, clinical applicability would be reserved for patients with T-cell deficiencies or for those patients infected with multidrug-resistant strains of *Mtb*. **(B)** Another approach to improve the initial phases of the immune response against *Mtb* is to explore novel ways of inducing vaccination to specifically activate NK cells. If vaccination could indeed induce proliferation and differentiation of memory NK cells that would create a subset of cells ready to act prior to encounter with *Mtb*, thereby enhancing innate immunity against the bacillus and perhaps, eventually prevent infection.

The adaptive-like qualities of NK cells make them new targets for the development of cellular-mediated therapies to enhance innate phases of the immune response against different pathogens and improve the protection conferred by currently available vaccines. Specifically, in the case of *Mtb* infection, one approach to achieve this purpose would be to prime NK cells with cytokines that augment function and allow improved responses against new antigen-dependent or -independent signals within the lung lesions of tuberculosis patients. An alternative approach would be to sensitize and expand NK cells using *in vitro* bacterial exposure, followed by autologous transfer of the sensitized cells back into to the infected individual. Although these cells would not act in the innate phase of infection, as patients are already infected, the NK cells may have enhanced function that would be of special importance in individuals with impaired T-cell responses. Finally, the fact that NK cells are a source of IL-22 during PTB provides rational support for the use of NK cells as a target of new vaccines to improve IL-22 production. This approach could provide patients with a cytokine involved in maintaining lung epithelium integrity from the early stages of the disease and help to limit bacterial dissemination (see Figure 2).

For years, efforts to understand why the human immune system cannot eradicate *Mtb* have focused on T-cell biology. However, the emerging role of NK cells during immunopathology of tuberculosis, and the antigen-specific recall responses of these cells, appeals for a redirection of attention to this lineage of lymphocytes which may represent “a new chance” against *Mtb*. Nevertheless, it is clear that much work remains to elucidate the molecular mechanisms, which regulate memory-like NK cell development and maintenance in response to *Mtb* infection. Also, immunologist has the task of ruling out possible collateral damage before implementing NK cells in protective strategies against *Mtb* and other pathogens since there are certain instances in which it has been possible to observe an association between the activity of NK cells with worsened outcomes and increased immunopathology (118–120). Nonetheless, current evidence does not support this scenario in the context of tuberculosis, as the data suggest that dysfunction of these cells is related with increased likelihood to become ill; in addition, there are discrepancies in the results of animal models and human studies of other infectious and non-infectious disorders that remain unclarified (121–123). Once such gaps in the knowledge have been overcome, the inherent memory-like qualities of this innate subset of cells may in fact be an old and well-known weapon, which could be used to further enhance innate phases of immunity against tuberculosis, improve vaccine design and efficacy, and perhaps, even to help to prevent infection.

## Author Contributions

All authors listed have made a substantial, direct, and intellectual contribution to the manuscript and approved it for publication.

## Conflict of Interest Statement

The authors declare that the research was conducted in the absence of any commercial or financial relationships that could be construed as a potential conflict of interest.
